# Counteracting action of *Bacillus stratosphericus* and *Staphylococcus succinus* strains against deleterious salt effects on *Zea mays* L.

**DOI:** 10.3389/fmicb.2023.1171980

**Published:** 2023-05-25

**Authors:** Gianmaria Oliva, Giovanni Vigliotta, Mattia Terzaghi, Francesco Guarino, Angela Cicatelli, Antonio Montagnoli, Stefano Castiglione

**Affiliations:** ^1^Department of Chemistry and Biology “A. Zambelli”, University of Salerno, Fisciano, SA, Italy; ^2^Department of Biosciences, Biotechnologies and Environment, University of Bari “Aldo Moro”, Bari, BA, Italy; ^3^Department of Biotechnologies and Life Sciences (DBSV), University of Insubria, Varese, Italy

**Keywords:** PGPR, soil salinization, antioxidant enzymes, ROS, seed coating, sustainable agriculture, root system

## Abstract

The salinization of soil is the process of progressive accumulation of salts such as sulfates, sodium, or chlorides into the soil. The increased level of salt has significant effects on glycophyte plants, such as rice, maize, and wheat, which are staple foods for the world's population. Consequently, it is important to develop biotechnologies that improve crops and clean up the soil. Among other remediation methods, there is an environmentally friendly approach to ameliorate the cultivation of glycophyte plants in saline soil, namely, the use of microorganisms tolerant to salt with growth-promoting features. Plant growth-promoting rhizobacteria (PGPR) can improve plant growth by colonizing their roots and playing a vital role in helping plants to establish and grow in nutrient-deficient conditions. Our research aimed to test *in vivo* halotolerant PGPR, isolated and characterized *in vitro* in a previous study conducted in our laboratory, inoculating them on maize seedlings to improve their growth in the presence of sodium chloride. The bacterial inoculation was performed using the seed-coating method, and the produced effects were evaluated by morphometric analysis, quantization of ion contents (sodium, potassium), produced biomass, both for epigeal (shoot) and hypogeal (root) organs, and by measuring salt-induced oxidative damage. The results showed an increase in biomass and sodium tolerance and even a reduction of oxidative stress in seedlings pretreated with a PGPR bacterial consortium (*Staphylococcus succinus* + *Bacillus stratosphericus*) over the control. Moreover, we observed that salt reduces growth and alters root system traits of maize seedlings, while bacterial treatment improves plant growth and partially restores the root architecture system in saline stress conditions. Therefore, the PGPR seed-coating or seedling treatment could be an effective strategy to enhance sustainable agriculture in saline soils due to the protection of the plants from their inhibitory effect.

## Graphical Abstract

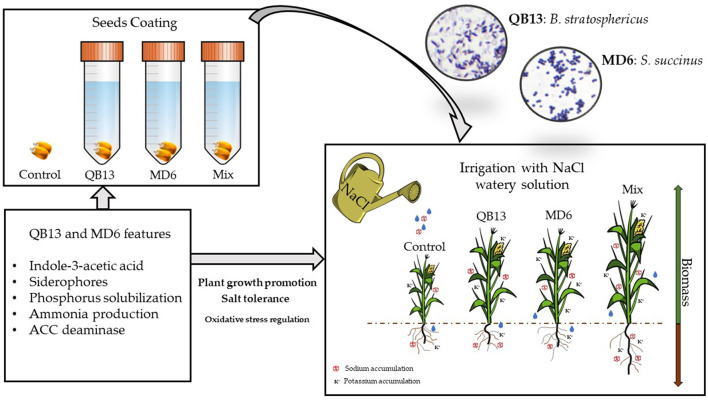

## 1. Introduction

Climate change will cause a significant loss of biodiversity, and it will be one of the greatest challenges our world has ever faced (Çolak et al., 2022). In fact, the increases in global average temperatures are compromising our climate, and these effects, if not counteracted with effective actions, are bound to worsen in the coming years. Climate change, therefore, represents a significant and lasting variation in the statistical distribution of weather trends and can occur over decades or even thousands of years (Harris, 2022). This change occurs in variations in the average weather conditions (i.e., a delay of the start of the wet season in the tropics), or in the extreme weather events frequency, such as floods, storms, and drought, which are increasingly evident. Long periods of drought, as well as the excessive exploitation of water resources and the irrigation of agricultural fields with low-quality water (e.g., salty water), can cause soil salinization. Soil salinity is a critical environmental factor that limits crop productivity since many of them are negatively affected by it (Machado and Serralheiro, 2017).

The beginning of the 21^st^ century has been characterized by the global reduction of water resources, environmental pollution, and an increase in salt concentration in soils and waters (Shahbaz and Ashraf, 2013). Every year, more and more agricultural lands are degraded by salinization, and this causes a significant decrease in crop productivity (Bennett et al., 2013). Based on these observations, it has been estimated that more than 50% of the cultivated land could deal with high salt concentrations by 2050 (Kumar and Sharma, 2020). Moreover, approximately 20% (45 million ha) of irrigated land is, at present, affected by salinization (Shrivastava and Kumar, 2015). The increased level of salt in the soil has significant effects on glycophyte plants such as rice, maize, or wheat, which are staple foods for the world's human population and livestock (Shah et al., 2017).

Saline soils can be classified into three classes based on salinity and sodium values, estimated by electrical conductivity (EC) and sodium adsorption ratio (SAR), or exchangeable sodium percentage (ESP): saline, saline sodium, and sodium (Poulopoulos and Inglezakis, 2016). Soil is defined as saline when the EC is slightly >4 deci-Siemens per meter (dS·m^−1^); mild between 4 and 8 dS·m^−1^; moderate between 8 and 16 dS·m^−1^; and high above 16 dS·m^−1^ (Sahab et al., 2021). The high salinity negatively affects the biochemical and physiological processes of plants (Giordano et al., 2021), such as the reduction of leaf surface, chlorophyll content, and the decrease in the photosynthetic efficiency of photosystem II (Kamran et al., 2020), and also inhibits plant growth by means of osmotic effects (Ma et al., 2020). Moreover, the root is the organ with the most direct exposure to adverse soil conditions, and its morphological plasticity is essential to withstand stress (Arif et al., 2019; Montagnoli et al., 2022). Salinity increases the proportions of fine roots improving the rhizosphere surface area so that plants can absorb more nutrients in low-nutrient soils (Wang et al., 2021). Consequently, the salinity alters different morphological and physiological traits of plants, modifying the root architecture system. Therefore, it is important to develop biotechnologies that enable the improvement of crop growth and the remediation of soils (Etesami and Maheshwari, 2018).

The approaches to improve saline soils are leaching, drainage systems, the application of specific chemicals and organic compounds, and the use of plants and microorganisms tolerant to salt (Orhan, 2021). Among the saline soil restoration methods, there is an environmentally friendly approach useful to ameliorate the cultivation of glycophyte plants, that is, the use of microorganisms tolerant to salt with growth-promoting features (Castiglione et al., 2021). Plant growth-promoting rhizobacteria (PGPR) improve plant growth by colonizing their roots and playing an essential role in helping plants to establish themselves and grow in nutrient-deficient conditions (Kumar et al., 2020). Their use in agricultural production could reduce agrochemicals and support environmentally friendly and sustainable food production. The demand for PGPR biofertilizers has risen day by day with the increasing importance of organic farming with minimal inputs of chemicals. The population of PGPR in the soil varies and largely depends on crop species and soil quality (Meena et al., 2020). PGPR promotes growth in three different ways: synthesizing hormones, facilitating the absorption of nutrients from the soil, and increasing tolerance to abiotic and biotic stresses. PGPR can (i) solubilize phosphates and mineralize other nutrients (Billah et al., 2019; Rawat et al., 2021), (ii) fix nitrogen (Aasfar et al., 2021), (iii) produce a class of hormones such as auxin (e.g., indole-3-acetic acid—IAA) (Myo et al., 2019; Grover et al., 2021), abscisic acid (ABA) (Haskett et al., 2021), gibberellin, and cytokinin (Vejan et al., 2016), and (iv) produce 1-aminocyclopropane-1-carboxylic acid (ACC) deaminase able to reduce the level of plant ethylene under stress conditions (Bomle et al., 2021; Chandwani and Amaresan, 2022). Moreover, some bacteria promote plant growth by improving root development and the consequent enhancement of water and mineral uptake (Tang et al., 2020), or by modifying its morphology (e.g., length and root diameter) (Rondina et al., 2020). A strategy for the application of PGPR in agriculture foresees their inoculation by seed coating.

The main aim of this study was to test *in vivo* halotolerant PGPR, isolated and characterized *in vitro* in a previous study (Castiglione et al., 2021). Therefore, we selected among our collection the bacterial strains showing plant growth-promoting (PGP) traits (e.g., production of ammonia, siderophores, and indole acetic-3-acid) and used the seed-coating method for their inoculation. Specifically, we chose the most promising gram-positive bacteria (*Bacillus stratosphericus* and *Staphylococcus succinus*) with the ability to grow in the presence of high concentrations of NaCl (>3 M). Finally, we evaluated PGP effects on the growth of glycophyte seedlings (maize) cultivated in a semi-hydroponic condition and exposed to saline stress (e.g., NaCl 75 mM).

## 2. Materials and methods

### 2.1. Strain characterization and culture conditions

The bacteria used in this study are the halotolerant/halophilic strains *S. succinus* MD6 and *B. stratosphericus* QB13, which were previously isolated from the rhizosphere of *Zea mays* L. and *Chenopodium quinoa* Willd., at the Plant Biology Laboratory of University of Salerno (Italy) and characterized about their growth-promoting features (Castiglione et al., 2021). For growth and propagation, bacteria were cultured on Luria-Bertani agar medium (g·L^−1^: tryptone 10.0, yeast extract 5.0, NaCl 10.0, and agar 15.0) at 28 ± 2°C.

### 2.2. Sterilization of seeds and coating

The bacteria treatment was carried out following the method described by Kaymak et al. (2009). Initially, maize seeds (cultivar DDK 7430, Dekalb, Italy) were sterilized with 70% ethanol incubating for 2 min at room temperature (RT) with constant shaking at 150 rpm and successively washed three times for 1 min with distilled sterile water. Finally, they were incubated for 20 min in 1.5% sodium hypochlorite at RT with constant shaking at 150 rpm and then rinsed with sterile distilled water. Seeds were soaked in a bacterial cell suspension of 0.1 optical density at 600 nm (OD600) (approximately 108 colony-forming units mL^−1^–CFU), prepared in physiological solution (0.9% NaCl) amended with 0.2% sucrose necessary to facilitate the adhesion of bacteria and incubated at RT, in a rotary shaker, at 100 rpm for 20 min. Before the seed coating, a microbial compatibility test was conducted between *B. stratosphericus* QB13 and *S. succinus* MD6, which highlighted no antagonistic action.

### 2.3. Experimental setup

Pretreated and untreated maize seeds were sown in sterile sand (autoclaved for 20 min at 120°C) in beaker pots of 0.6 L. Initially, these pots were placed in a climatic room at a temperature of 24°C and 70% relative humidity for seed germination and successive seedling acclimation. After 7 days, seedlings were transferred to a greenhouse setup with a photoperiod of 16 h of light and 8 h of dark and at a constant temperature of 21°C. Eight experimental treatments (three plants for each treatment) were set up: a group of four not exposed to saline treatment and another group exposed to NaCl. The first group was irrigated with 50 mL of Hoagland's solution (Hoagland and Arnon, 1950), every 2–3 days, while the other group was irrigated with 75 mM NaCl Hoagland's solution, until a total salt administration of 30 mmol was reached. For each group of four, one treatment was in the absence of bacteria (control), and the remaining ones were treated with *B. stratosphericus* QB13, *S. succinus* MD6, and a mixture (*B. stratosphericus* QB13 + *S. succinus* MD6), respectively. The choice of the 75 mM NaCl concentration was determined by a preliminary dose/toxicity evaluation of the salt on the maize seedlings. Among the different tested concentrations (15–100 mM NaCl), the maize seedlings showed, at 75 mM, the greatest phenotypical effects on growth, remaining still viable.

### 2.4. Seedling growth and biomass determination

A week after the last irrigation, plants were removed from beaker pots, and the stem length of each seedling was measured. The epigeal part (leaves plus stem) and the roots were collected separately, sand particles were carefully removed from the roots, and the fresh biomass was measured. Then, samples were dried at 80°C until constant weight, for dry mass determination.

### 2.5. Root morphological measurements

Maize sand-free roots were scanned submerged in water at a resolution of 800 dpi with a calibrated flatbed scanner coupled to a lighting system for image acquisition (Epson Expression 12000 XL). Images were analyzed by WinRhizo Pro V. 2007d (Regent Instruments Inc. Quebec) by micrometric image analysis as described in a study by Montagnoli et al. (2018). This method analyzes each image with a progressive increment of 50-μm diameter units. By analyzing the color histogram representing the data of fine root length (mm) for each 50-μm-diameter unit and observing how these units distribute within the scanned images, it was possible to group them into three diameter classes as follows: very fine roots, d < 0.2 mm; fine roots, 0.2 < d < 0.85 mm; and thick roots, d >0.85 mm. The maximum diameter measured in the last root class was 3.8 mm. Root length was calculated according to the new classes by summing the length values of the 50-μm diameter units falling within each diameter class.

### 2.6. Sodium and potassium concentrations

The concentrations of Na^+^ and K^+^ were determined by atomic absorption spectroscopy (Perkin Elmer AAnalyst 100 CV-AAS, Wellesley, MA, USA). The standard curve per ion quantitation was prepared by mixing nine different concentrations of NaCl (Sigma-Aldrich, Milano, Italy) or KCl (Pokler Italia, Salerno, Italy) ranging between 0.1 and 10.0 mg·L^−1^ (correlation factor R^2^ ≥ 0.97). The dried biomass (root and epigeal part) was manually pulverized in mortars using liquid nitrogen. Afterward, 250 mg of each sample was mineralized in a microwave (Milestone Ethos Shelton, CT, USA), using 4 mL of 65% HNO_3_ and 2 mL of 50% HF. A six-step mineralization program was employed: 250 W for 2 min, 0 W for 2 min, 250 W for 5 min, 400 W for 5 min, 0 W for 2 min, and 500 W for 5 min (Baldantoni et al., 2019). Subsequently, the solutions were diluted to a final volume of 50 mL, and then, ion concentrations were determined.

### 2.7. Hydrogen peroxide determination

The H_2_O_2_ levels were estimated according to the study by Velikova et al. (2000). Leaf tissues (0.1 g) were pulverized into liquid nitrogen and then homogenized by IKA T10 basic homogenizer at maximum speed setting in an ice bath with 1.0 mL 0.1% (w/v) trichloroacetic acid (TCA). The homogenate was centrifugated at 13,000 *g* at 4°C for 15 min. Then, 500 μL of supernatant was mixed with 500 μL of 10 mM (pH 7.0) potassium phosphate buffer and 1.0 mL of 1.0 M KI. The levels of H_2_O_2_ were quantified spectrophotometrically at 390 nm (Shimadzu UV-1800, Milano, Italy).

### 2.8. Malondialdehyde quantification

First and foremost, leaf tissues (0.1 g) were pulverized in liquid nitrogen and then homogenized at maximum speed setting in an ice bath with 1.0 mL 0.1% (w/v) TCA. The homogenate was centrifugated at 13,000 g at 4°C for 15 min. Afterward, 400 μL of supernatant was added to 1.0 mL premixed solution of TCA 20% (w/v) and thiobarbituric acid (TBA) 0.5% (w/v). Then, samples were incubated at 95°C for 30 min in the dark. At the end of the incubation period, the reaction was stopped in an ice bath, the absorbance was measured spectrophotometrically at 532 nm, and the value, for non-specific absorption, at 600 nm was subtracted. Finally, the amount of malondialdehyde (MDA) was calculated from the extinction coefficient 155 mM^−1^ cm^−1^.

### 2.9. Catalase activity

Catalase enzyme activity was estimated according to the study by Shahzadi et al. (2021). Leaf tissues (0.25 g) were pulverized into liquid nitrogen and then homogenized in 4.0 mL of 0.2 M (pH 7.8) potassium phosphate supplemented with 0.1 mM EDTA. The homogenate was centrifugated at 4°C at 13,000 g for 15 min. Next, 100 μL of supernatant was mixed with 2.8 mL of 50 mM (pH 7.0) potassium phosphate buffer and 100 μL of 300 mM H_2_O_2_. Finally, the absorbance was measured at 240 nm.

### 2.10. Statistical analysis

The stem length and biomass, the sodium amount of the seedlings, and the root traits were statistically analyzed by a two-way ANOVA to test the effect of the independent factors (salt and bacteria treatments). When needed, the dependent variables were square root or log-transformed to ensure normal distributions and equal variances. A *post-hoc* LSD or Bonferroni test was conducted at a p-value of < 0.05. Statistical analysis was carried out using the statistical software package SPSS 25.0 (SPSS Inc., Chicago, IL, United States).

## 3. Results

### 3.1. Plants growth and biomass

Seedlings exposed to NaCl, regardless of seeds pretreated with bacteria, as expected showed a lower growth than those untreated with salt, with an evident reduction in biomass and stem length (Table 1). The treatments with bacteria were specifically associated with further biomass variations; in fact, the co-infection of the seeds with *S. succinus* MD6 and *B. stratosphericus* QB13 resulted in a significant biomass increase compared to control plants, approximately 60 and 85% in the fresh and dry weight of roots, respectively, and 67 and 51% in epigeal parts (*p* < 0.05) in plants exposed to saline stress. Treatment with *B. stratosphericus* QB13 or *S. succinus* MD6 alone did not result in statistically significant changes, although mean dry or fresh weight values were slightly higher. With no addition of NaCl, except for *S. succinus* MD6, there were no statistically significant changes in both the roots and epigeal parts. However, with *B. stratosphericus* QB13, a positive trend was observed. Seedlings from pretreatment with *S. succinus* MD6 alone showed a reduction in mean weight values in the epigeal parts (fresh weight, *p* < 0.05), but in seedlings with *B. stratosphericus* QB13 co-infection, this effect was not evident or strongly reduced, in accordance with the positive trend of biomass production associated with the latter treatment.

**Table 1:** Biomass and stem length.

**Treatment**	**Salt solution (mmol NaCl)**	**Stem length [cm]**	**Root fresh weight [g]**	**Root dry weight [g]**	**Epigeal part fresh weight [g]**	**Epigeal part dry weight [g]**
Control	0	88.7 ± 9.7	29.44 ± 4.61^ab^	2.53 ± 0.46	125.77 ± 13.51^a^	11.95 ± 2.40^ab^
	30	34.5 ± 4.2	10.02 ± 2.45^y^	0.39 ± 0.07^y^	16.27 ± 3.50^y^	2.22 ± 0.60^y^
QB13	0	82.0 ± 6.2	39.46 ± 3.48^a^	3.11 ± 0.44	134.11 ± 6.23^a^	14.17 ± 0.78^a^
	30	44.8 ± 3.6	10.91 ± 4.87^xy^	0.57 ± 0.16^xy^	19.16 ± 4.79^xy^	2.24 ± 0.75^xy^
MD6	0	72.3 ± 4.9	25.19 ± 3.00^b^	2.48 ± 0.79	94.34 ± 12.06 b	9.38 ± 1.44 b
	30	35.7 ± 8.0	11.77 ± 1.51^xy^	0.68 ± 0.03^xy^	21.60 ± 1.31^xy^	2.46 ± 0.11^xy^
Mix	0	75.3 ± 5.5	35.78 ± 6.45^ab^	2.99 ± 0.75	113.71 ± 6.57^ab^	11.42 ± 1.75^ab^
	30	37.7 ± 7.8	16.44 ± 1.35^x^	0.72 ± 0.07^x^	27.12 ± 3.70^x^	3.35 ± 0.47^x^

Values are the means of three replicates. ^a, b, c^Indicate significant differences among microbial treatments in plants irrigated without salt (LSD test, *p* < 0.05). ^x, y, z^Indicate significant differences among microbial treatments in plants exposed to NaCl (LSD test, *p* < 0.05). The absence of letters indicates no statistical significance. QB13: *B*. *stratosphericus*, MD6: *S. succinus*, and Mix: treatment with both bacteria.

**Figure 1:**
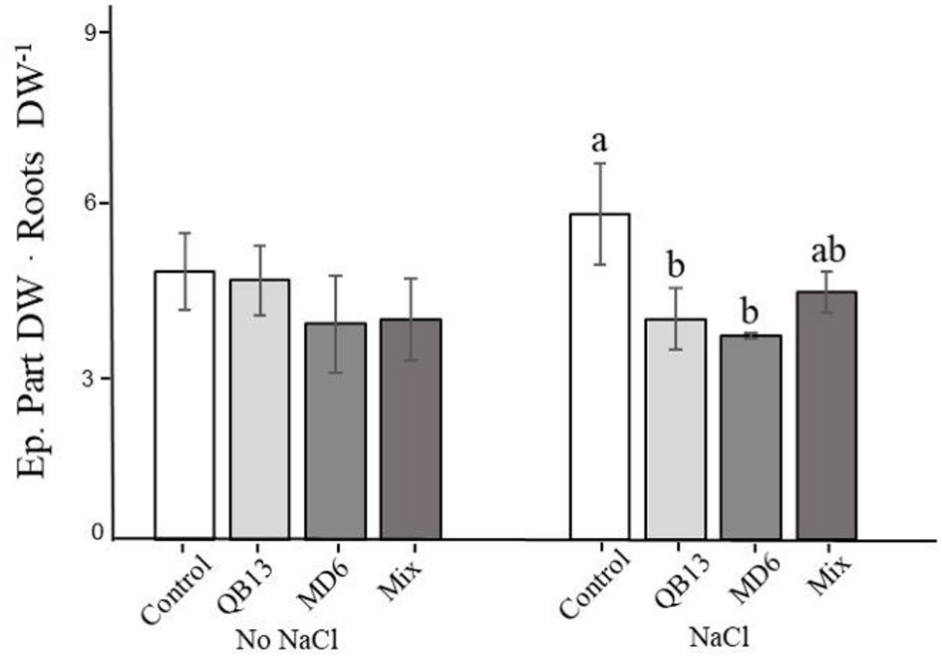
Epigeal part dry weight/roots dry weight ratio. Values are the means of three replicates. a, b, and c indicate significant differences among microbial treatments (LSD test, *p* < 0.05). The absence of letters indicates no statistical significance. QB13: *B. stratosphericus*, MD6: *S. succinus*, and Mix: treatment with both bacteria.

Further biomass analyses showed that the growth in the presence of salt resulted in an increase in the ratio of epigeal parts to roots by 20% (*p* < 0.05) (Figure 1). This variation was not highlighted in plants pretreated singularly with *B. stratosphericus* QB13 or *S. succinus* MD6, in fact, values were similar to those of the samples grown with no NaCl. This positive result was less evident in plants deriving from seeds co-infected with the Mix, probably due to the effect on the biomass increase in both roots and epigeal parts (Table 1). Compared to the control, the absence of salt pretreatment with bacteria was not associated with significant changes in this ratio (Figure 1).

### 3.2. Analysis of root system traits

**Figure 2:**
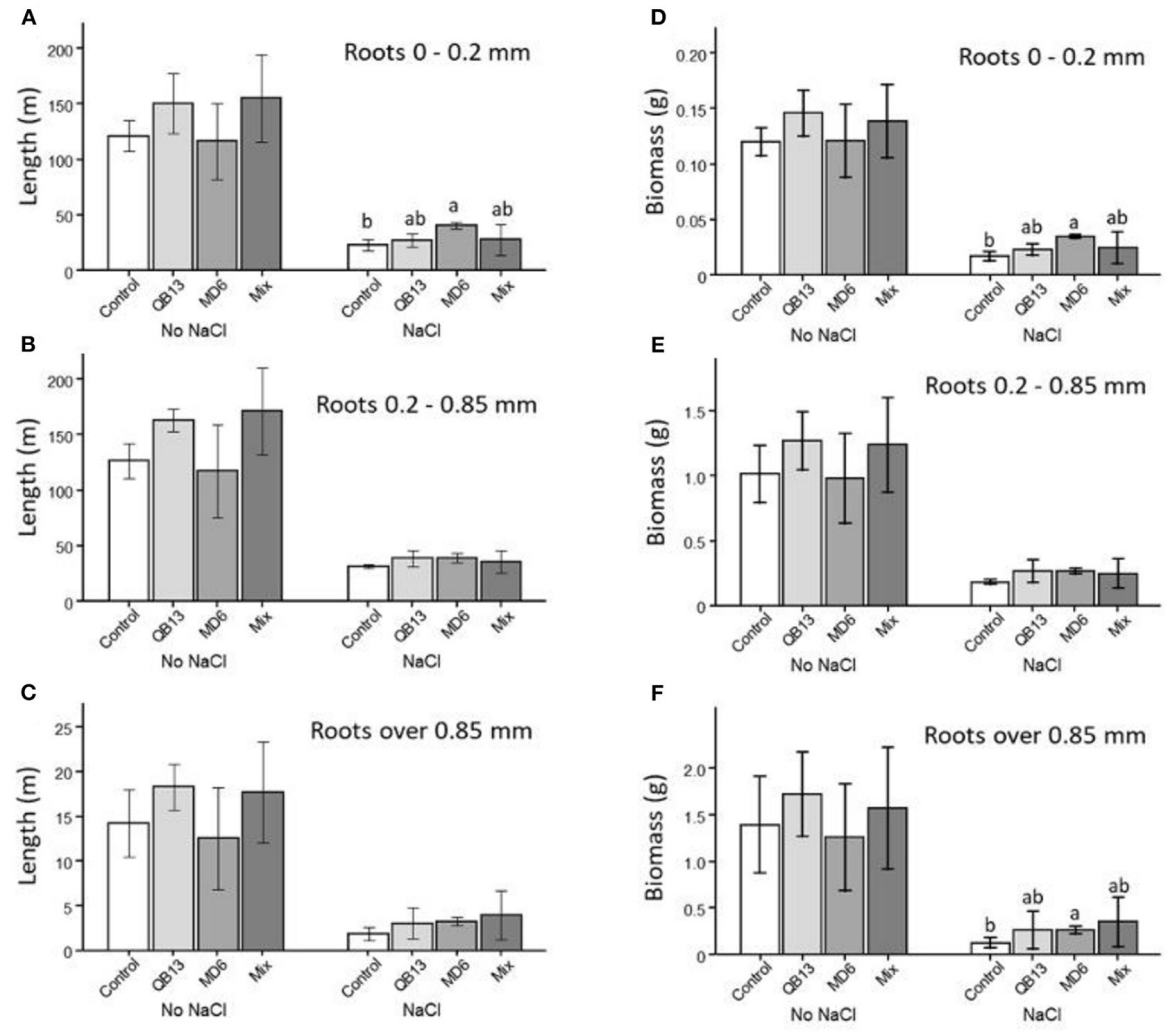
**(A–C)** Total root length for the three diameter classes: very fine <0.2mm; fine 0.2–0.85mm; and thick >0.85mm. **(D–F)** Total root biomass for the three diameter classes: very fine <0.2mm; fine 0.2–0.85mm; and thick >0.85mm. The letters a and b indicate significant differences (LSD test, *p* < 0.05) among microbial treatments, and the absence of letters indicates no statistical significance. QB13: *B. stratosphericus*, MD6: *S. succinus*, and Mix: treatment with both bacteria.

**Figure 3:**
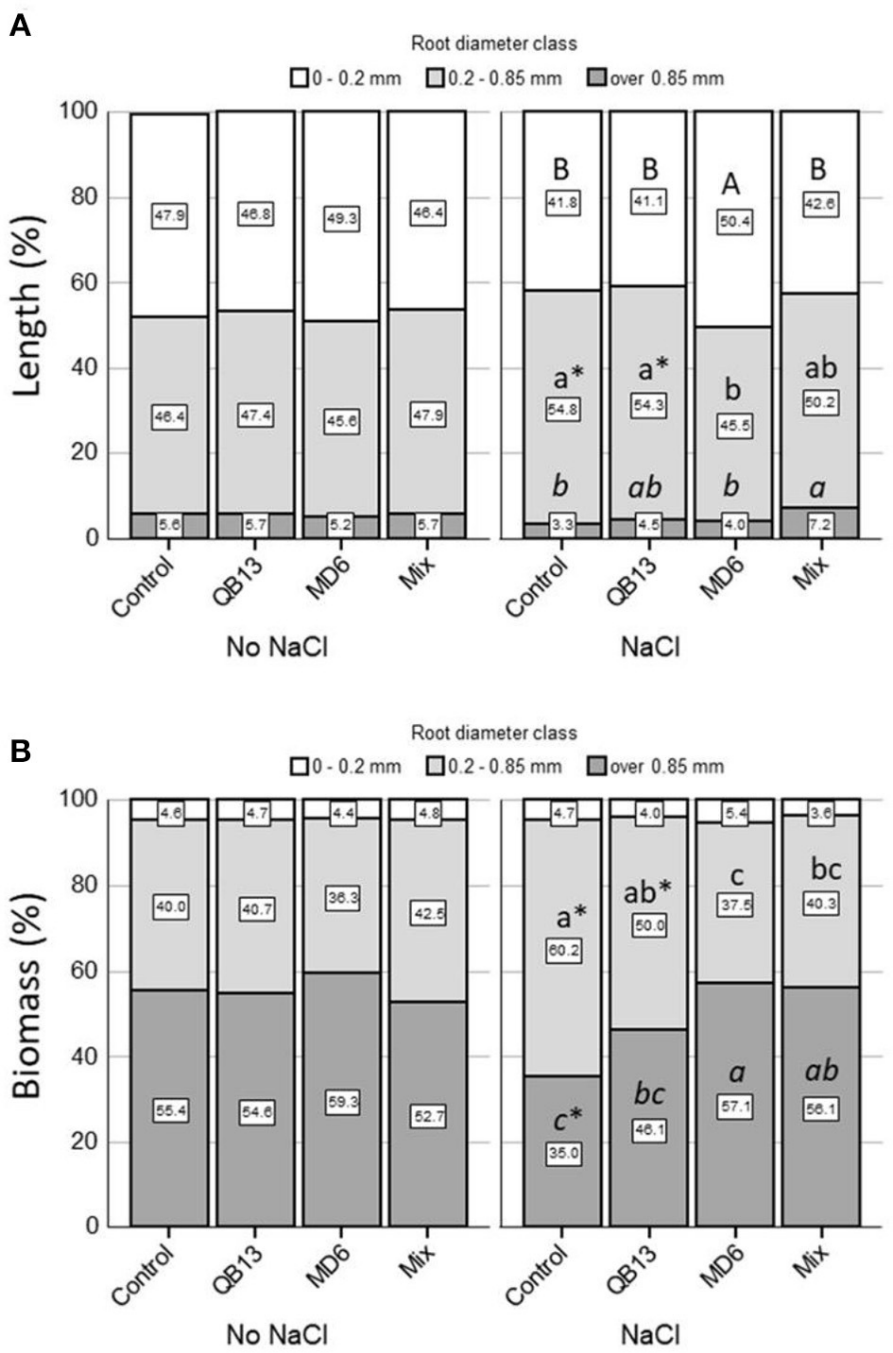
**(A)** The Root length percentage and **(B)** the root biomass percentage of the three diameter classes. The very fine root class (<0.2mm) is represented in white, the fine root class (0.2–0.85mm) in light gray, and the thick root class (>0.85mm) in dark gray. Letters compare data among bacterial treatments within each root diameter class and salt treatment. An asterisk ^*^indicates a significant difference (LSD test, *p* < 0.05) between salt treatments (No NaCl, NaCl) within each root diameter class and bacterial treatment. The absence of letters or ^*^ indicates no statistical significance. QB13: *B. stratosphericus*, MD6: *S. succinus*, and Mix: treatment with both bacteria.

A further evaluation was performed by considering the effects of the treatments on the root system traits, focusing on the length and weight of the three diameter classes: very fine <0.2 mm, fine 0.2–0.85 mm, and thick >0.85 mm (Figures 2, 3). Both in the absence and in the presence of saline solution, approximately 95% of the total length was determined by very fine roots and fine roots (Figures 2A–C, 3A). In seedlings grown in the absence of salt, the overall length and the relative distribution of the classes were similar between controls and samples pretreated with bacteria. In fact, very fine and fine roots were prevalent, both with a similar frequency of 45–48% (Figures 2A–C, 3A). In the presence of NaCl, the total length of all root classes resulted reduced by more than 75% (Figures 2A–C). In the control seedlings, the 0.2–0.85 fine roots fraction was significantly greater than in the absence of salt treatment by approximately 18% (*p* < 0.05) (Figure 3A). Compared to the control, the pretreatment with *B. stratosphericus* QB13 was not associated with significant modifications. On the contrary, the one pretreated with *S. succinus* MD6 showed an increase by approximately 70% in the total length of the very fine roots, resulting in an increase in the total root system length and in a restoring of the relative diameter class distribution to the values found for seedlings grown under no salt addition (Figures 2A–C, 3A). Seedlings deriving from seeds pretreated with the Mix did not show the same differences in the case of the *S. succinus* MD6 pretreatment; however, the percentage of thick roots result was a significant increase of more than 2-fold (*p* < 0.05).

A comparative analysis of the biomass showed that under all conditions the total root system weight was determined (more than 95%) by the 0.2–0.85 and >0.85 mm root classes (Figures 2D–F, 3B). The effects associated with salt and bacteria were consistent with those observed for the root length. In the absence of salt, the total weight of all roots and their relative distribution across the three diameter classes were similar between controls and pretreated seedlings. In these conditions, the fine and thick fine root classes contributed, on average, 40 and 55%, respectively (Figures 2D–F, 3B). The addition of NaCl caused a reduction of 80–85% of the total weight of all root classes and an inversion of the percentages of the two prevalent classes (0.2–0.85 and >0.85), which were, on average, 60 and 35%, respectively (Control, Figure 3B). This latter observation was not highlighted in seedlings pretreated with *S. succinus* MD6 alone, and with the Mix, where weight distribution was similar to that of no salt addition (*p* < 0.05).

### 3.3. Na^+^ and K^+^ quantification and distribution

All seedlings grown in the presence of NaCl showed an accumulation of Na^+^ ions in root and epigeal parts, and in particular, the bacterial pretreatment resulted in a further increase. Compared to control samples grown with no salt added, the salt concentration increases varied from 4 to 6 times in the roots, and from 45 to, approximately, 90 times in the epigeal parts, without or with bacterial pretreatment, respectively (Table 2). Compared to controls, in saline conditions, pretreatment with *B. stratosphericus* QB13 resulted in an accumulation increase of approximately 27% in the roots (64.57 ± 2.71 vs. 50.97 ± 1.16 mg·g^−1^ DW) (*p* < 0.05) but not in epigeal parts. On the contrary, the *S. succinus* MD6 pretreatment had the opposite behavior, with a 28% of Na^+^ increment in epigeal parts (27.05 ± 0.51 vs. 21.12 ± 3.81) (*p* < 0.05), and with not a significant effect in the roots. Co-infection of the seeds with the Mix resulted in a complementary and more helpful effect, with an increment of 38% in the roots and 88% in the epigeal parts (70.67 ± 2.39 vs. 50.97 ± 1.16 mg·g^−1^ DW, and 39.66 ± 1.42 vs. 21.12 ± 3.81 mg·g^−1^ DW, respectively - *p* < 0.05), that is, a further 10% increase in the roots and 45% in the epigeal parts, compared to an effective action evidenced for the bacteria added singularly. Moreover, the NaCl treatment improved the K^+^ content in the epigeal parts. In the controls of the unpretreated seedlings (0 and 30 mmol), the K^+^ concentrations increased by approximately 25%. Similarly to the Na^+^ accumulation, the bacterial treatment improved the K^+^ accumulation by approximately 30% (Table 2).

**Table 2:** Effect of bacteria on epigeal part and root nutrient uptake (mg·g^−1^ dry weight) of maize irrigated with salt solution.

**Treatment**	**[mmol NaCl]**	**[Na^+^] in Roots**	**[Na^+^] in Epigeal part**	**[K^+^] in Roots**	**[K^+^] in Epigeal part**
Control	0	11.21 ± 0.76^a^	0.46 ± 0.03	33.03 ± 6.52	49.62 ± 7.69
	30	50.97 ± 1.16^z^	21.12 ± 3.81^z^	30.65 ± 3.89	64.81 ± 8.32^z^
QB13	0	6.34 ± 0.65^b^	0.44 ± 0.03	31.14 ± 5.86	51.97 ± 1.59
	30	64.57 ± 2.71^y^	24.91 ± 0.73^yz^	35.51 ± 2.57	80.62 ± 5.20^y^
MD6	0	9.20 ± 1.39^a^	0.47 ± 0.01	34.52 ± 2.60	49.39 ± 3.97
	30	57.02 ± 6.45^yz^	27.05 ± 0.51^y^	17.65 ± 2.00	93.40 ± 2.85^x^
Mix	0	18.71 ± 1.52^a^	0.51 ± 0.06	23.93 ± 1.84	49.61 ± 2.07
	30	70.67 ± 2.39^x^	39.66 ± 1.42^x^	37.94 ± 18.73	80.96 ± 2.43^y^

Values are the means of three replicates. ^a, b, c^Indicate significant differences among microbial treatments within nutrient uptake (Bonferroni test, *p* < 0.05). ^x, y, z^Indicate significant differences among microbial treatments within nutrient uptake and saline treatments (Bonferroni test, *p* < 0.05). The absence of letters indicates no statistical significance. QB13: *B. stratosphericus*, MD6: *S. succinus*, and Mix: treatment with both bacteria.

**Figure 4:**
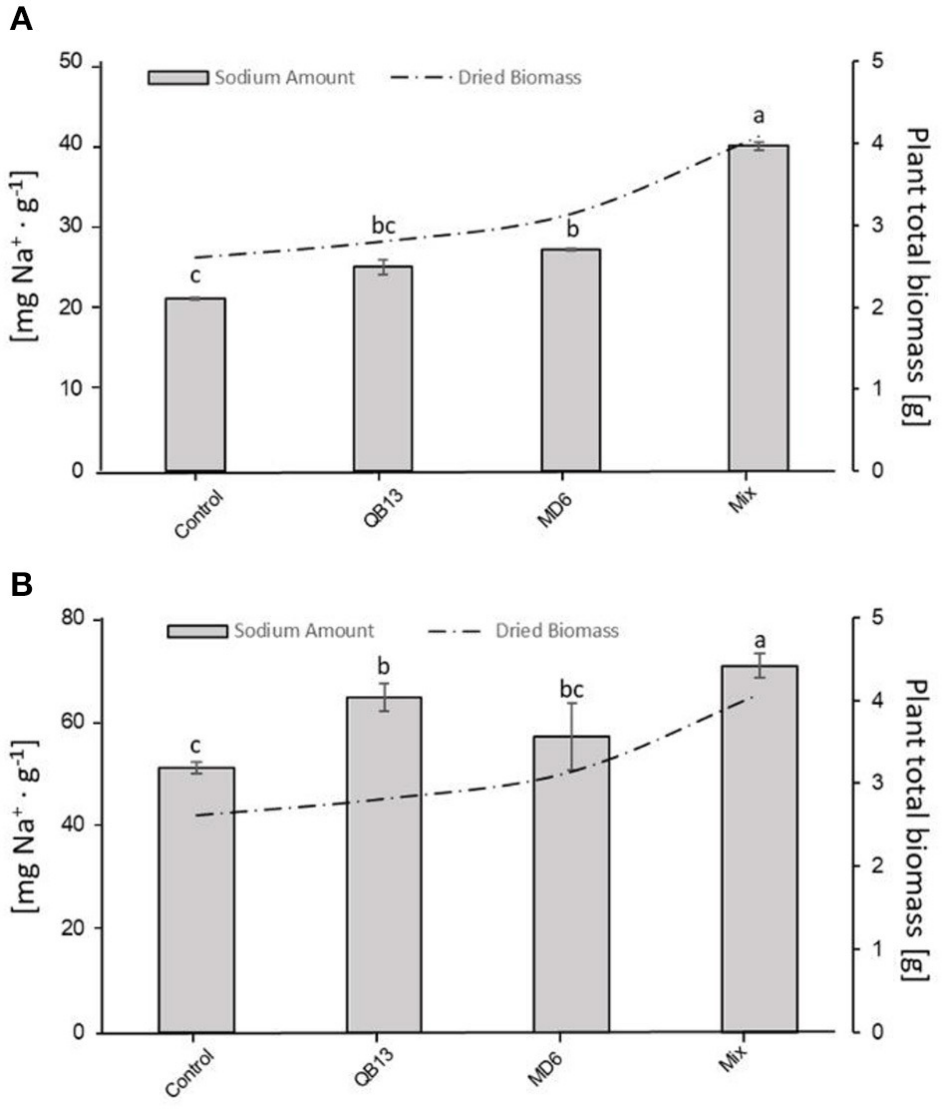
Correlation between Na^+^ amount in epigeal part (A) or roots (B) and total biomass (roots plus epigeal parts) produced by plants exposed to NaCl. The letters a, b, and c indicate significant differences (LSD test, *p* < 0.05) among microbial treatments. The absence of letters indicates no statistical significance. QB13: *B. stratosphericus*, MD6: *S. succinus*, and Mix: treatment with both bacteria.

The Na^+^ accumulation and biomass production, in the seedlings grown in saline conditions (Tables 1, 2), were compared to each other (Figure 4). In fact, there was a significant correlation between the increase in Na^+^ in both roots and epigeal parts and the total biomass (dry weight) in the pretreated seedlings (*p* < 0.05). In the Mix, where the effects were more evident, the total biomass and the total Na^+^ accumulation were both an average of 64% greater than in the uninoculated seedlings (Figure 4, Tables 1, 2). Hence, the pretreated seedlings with the Mix exhibited a reduced sensitivity to the salt, evidenced by an improved Na^+^ accumulation as well as greater growth.

### 3.4. Effect of NaCl on oxidative damage

**Figure 5:**
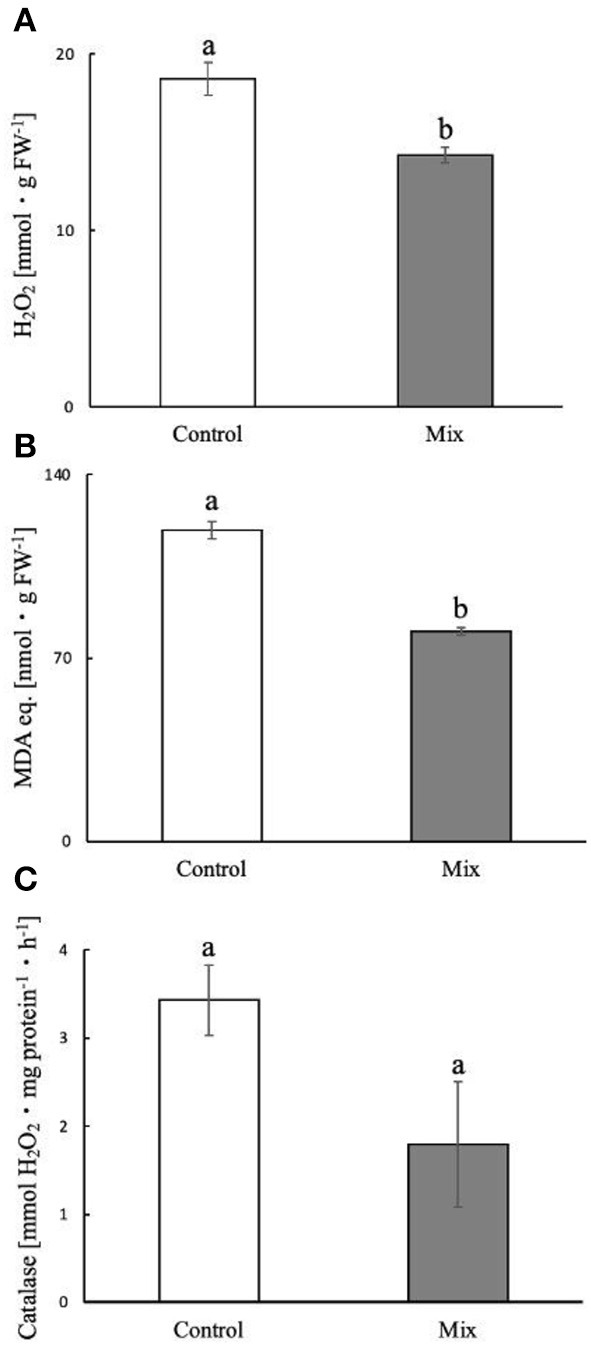
Amount estimation of hydrogen peroxide **(A)**, malondialdehyde **(B)**, and catalase activity **(C)** in maize leaves exposed to salt. The letters a and b indicate significant differences (Tukey HSD test, *p* < 0.05) among control and microbial treatment. Mix: treatment with both bacteria.

Salt stress induces oxidative damage in all organisms, and plant salt tolerance has been related to the ability to counteract these damaging effects. To gain insight into possible mechanisms of NaCl tolerance, we focused on maize seedlings pretreated with bacterial Mix, which resulted in a more tolerance to salt. Afterward, we examined the levels of hydrogen peroxide, an indicator of the presence, at the cellular level, of reactive oxygen species (ROS), the damage to the membrane lipids, testing the concentrations of MDA, and finally, the activity of the H_2_O_2_ scavenging of catalase (CAT) enzyme. We observed (Figures 5A, B) that the concentrations of hydrogen peroxide and MDA decreased significantly (*p* < 0.05) by approximately 23% (18.58 ± 0.92 vs. 14.27 ± 0.45 mmol·g FW^−1^) and 33% (118.87 ± 3.38 vs. 80.16 ± 1.48 nmol·g FW^−1^), respectively, in the leaves of the seedlings exposed to salt and pre-inoculated with Mix compared to the control. The mean catalase activity (Figure 5C) was 47% lower than the control, although the difference was not significant (*p* > 0.05).

## 4. Discussion

Soil salinity is a widespread phenomenon in developing countries and in intensively exploited agricultural lands. This causes the loss of 20–50% of agricultural productivity, limiting plant growth and yield (Shokat and Großkinsky, 2019). Therefore, it is important to look for new biotechnologies to improve the growth and tolerance of crops grown in saline soils (Egamberdieva et al., 2019). An effective strategy to counteract this phenomenon could be the exploitation of halotolerant PGPR, since these microorganisms may improve crop development and agricultural productivity in conditions of saline stress (Nawaz et al., 2020). PGPR stimulates plant growth by direct or indirect mechanisms. According to our previous study (Castiglione et al., 2021) in which we highlighted *B. stratosphericus* QB13 and *S. succinus* MD6 PGPR features (as IAA, siderophores production), Siddikee et al. (2010) have described that some strains of B. stratosphericus, a halotolerant bacterium, can fix nitrogen, produce IAA, solubilize P and Zn, oxidize S, and produce NH_3_, promoting plant growth under conditions of saline stress. Khan et al. (2020) observed the role of *B. stratosphericus* LW-03 in antifungal activity against common plant pathogens and its ability to exhibit multiple plant growth-promoting traits, including ACC deaminase activity and IAA production. Orhan and Demirci (2020) described that *S. succinus* exhibited some PGP traits as nitrogen fixation and ammonia production. Moreover, several strains of this species produce IAA, siderophore, and ACC deaminase (Mayak et al., 2004; Cheng et al., 2007). Therefore, seed-coating or seedling inoculation with *B. stratosphericus* and/or *S. succinus* could improve the growth and tolerance of glycophyte plants cultivated in saline soils (Ma, 2019). In fact, in our experiment, we observed a significant reduction in stem length and total plant biomass when exposed to NaCl. However, the Mix treatment (*B. stratosphericus* plus *S. succinus*) improved the epigeal and root biomass, while pretreatment with a single bacterium showed a decrease in the epigeal/roots biomass ratio. Olanrewaju et al. (2017) reported that bacteria, able to produce ACC deaminase, could reduce ethylene stress and promote plant growth, while Keswani et al. (2020) showed that the production of IAA could improve both root development and plant growth. Therefore, the improvements induced by our bacteria might be explained by IAA production and ACC deaminase activity being able to reduce the ethylene stress by implementing root development and plant growth (Ha-Tran et al., 2021). Finally, the decrease in epigeal and root biomass ratio under NaCl treatment could be associated with an improvement in salt tolerance; in fact, an increase in functional root biomass has been reported to strengthen the root Na^+^ detoxification capability (Bano and Fatima, 2009).

Moreover, we highlighted how saline stress influenced and modified the root system traits, reducing both its length and biomass. Likewise, An et al. (2003) showed the same effect of saline stress on soybean crops. In our experiment, salt modifies the root system by increasing the length and weight of the fine root diameter class (0.2–0.85 mm) compared to the thicker one. In maize seedlings exposed to salt and pre-infected with *S. succinus* and, to a lesser extent, those pre-infectes to the Mix, the root traits showed a very similar distribution to the untreated seedlings. Moreover, we have shown that *S. succinus* promotes the production of very fine roots (< 0.2 mm). Some authors showed that PGPR, such as *Bacillus amyloliquefaciens*, stimulates root development by increasing its total length as well as the production of finer roots (Irizarry and White, 2017). In addition, root systems constituted by very fine roots develop larger contact surfaces to uptake nutrients at lower relative carbon costs. The greater root surface determines a more efficient water and nutrient uptake (German et al., 2000). Hence, *S. succinus* MD6 could contribute to the protection of the root system from salt stress, preserving its functionality.

Our data show how the bacterial seed pretreatment not only promotes crop growth under salt conditions but also the uptake and storage of Na^+^, especially in the Mix pretreated seedlings. Compared to the control, the greatest accumulation of Na^+^, without both reduction of biomass and alteration of seedling morphological parameters, could be favored by the synergistic or additive effect of the Mix. Indeed, according to Ullah et al. (2021), some bacteria (e.g., *Pseudomonas putida* and *Bacillus* spp.) significantly increase the content of Na^+^ and some nutrients in the roots and epigeal parts. This higher uptake of nutrients could be justified by greater solubilization, in the rhizosphere, of unavailable forms of these nutrients in saline-sodic soils. Moreover, we also detected an increase in K^+^ in epigeal parts in the pretreated seedlings irrigated with NaCl, which could explain better osmoregulation. According to Maayhuis and Amtmann (2004), the capability, to counteract salinity stress, strongly depends on K^+^ availability. In fact, under salinity stress, NaCl induced a rapid K^+^ loss from the cytosol that interferes with its homeostasis. Thus, an important strategy for plant salt tolerance could be its ability to retain K^+^ under salt stress (Wu et al., 2018). Moreover, we observed a significant increase of K^+^ in epigeal parts exposed to saline treatment, demonstrating how PGPR can induce tolerance in seedlings against NaCl stress, maintaining a greater amount of K^+^ in order to preserve a higher K^+^/Na^+^ ratio (Rojas-Tapias et al., 2012). In plants, salt stress induces the production of ROS, which causes molecular damage and impairment of cellular functions. Several strategies have been described to counteract the production of ROS, such as upregulation of the activities of antioxidant enzymes, CAT, peroxidase (POD), and superoxide dismutase (SOD) (Kang et al., 2014), and, for salt-tolerant plants, the reduction of their generation by downregulating the electron transport chain (ETR) at the level of photosynthetic systems (Bano et al., 2021). We observed that the pretreatment with bacterial Mix in seedlings exposed to NaCl resulted in a significant reduction of the levels of H_2_O_2_ and MDA when compared to the unpretreated samples.

In summary, we demonstrated *in vivo* that halotolerant strains of *B. stratosphericus* QB13 and *S. succinus* MD6 exhibit PGP properties, and can promote the development of maize seedlings at harmful salt concentrations. Here, we analyzed the effects of NaCl on plant growth, biomass production, Na^+^ and K^+^ accumulation, root system, and oxidative stress and also evaluated the capability of bacteria to interact with these processes. We reported that the isolated strains can effectively reduce the sensitivity to salt stress by various mechanisms, such as the control of K^+^/Na^+^ balance, oxidative stress, and root system development. Singularly, both bacteria can act on the same process and in a strain-dependent manner (effect on epigeal/roots dry weight ratio, K^+^ and Na^+^ accumulation), or specifically on different processes. All the effects found in the treatments with the single strain are highlighted in the Mix pretreated seedlings. Some of them are enhanced (Na^+^ accumulation), while others are induced exclusively by the bacterial Mix as biomass production. These differences, as well as other factors resulting from their interaction, could underlie the greater reduction in salt sensitivity observed in maize seedlings treated with both microorganisms.

## 5. Conclusion

The high soil salinity induces functional and morphological alterations of plants (e.g., growth, development of leaf surface, chlorophyll content, photosynthetic efficiency, and root architecture system) through mechanisms that are still poorly understood, including osmotic imbalance and induction of oxidative stress. We reported here the results that show the capability of two PGPR strains to counteract the NaCl deleterious effect on the glycophyte seedlings of maize, favoring their development at harmful concentrations.

We conclude that the use of *B. stratosphericus* QB13 and *S. succinus* MD6, in combination with the most common restoration methods (e.g., percolation, drainage systems, and application of chemical compounds), or as an alternative to them, could represent an effective environmentally friendly approach to make degraded soils suitable for agricultural purposes. In addition, other aspects could make the two bacterial strains interesting from a biotechnological perspective. In fact, due to their tolerance to salt and the ability to favor the accumulation of Na^+^ in the maize seedlings, they could have potentially useful features in bioremediation practices for the control of the amounts of this ion, or other toxic compounds present in the saline soils.

However, further studies will aim to better characterize the mechanisms in which the two new PGPR strains act for the regulation of salt tolerance, and their actions on other development processes of the maize plant (e.g., inflorescence and caryopsis production), including investigating their potential in further *green technology* applications.

## Data availability statement

The raw data supporting the conclusions of this article will be made available by the authors, without undue reservation.

## Author contributions

SC, GV, GO, and MT: conceptualization. GO, MT, and GV: methodology, writing—original draft preparation, and visualization. GO, FG, and MT: software. GO, GV, MT, AC, FG, and SC: investigation, data curation, and writing—reviewing and editing. SC and AM: resources. AM: supervision and project administration: Department of Chemistry and Biology-University of Salerno. SC, MT, GV, AC, and FG: funding acquisition. All authors have read and agreed to the published version of the manuscript.
